# Identification of epigallocatechin-3-*O*-(3-*O*-methyl)-gallate (EGCG3′′Me) and amino acid profiles in various tea (*Camellia sinensis* L.) cultivars

**DOI:** 10.1016/j.dib.2017.08.007

**Published:** 2017-08-10

**Authors:** Hyang-Gi Ji, Yeong-Ran Lee, Min-Seuk Lee, Kyeng Hwan Hwang, Eun-Hee Kim, Jun Seong Park, Young-Shick Hong

**Affiliations:** a Division of Food and Nutrition, Chonnam National University, Yongbong-ro, Buk-gu, Gwangju 500-757, Republic of Korea; b Applied Technology & Research Division, R&D Center, AmorePacific Corporation, Yongin-si, Gyeonggi-do 446-729, Republic of Korea; c Osulloc Tea R&D Center, Osulloc Farm Corporation, Jeju 699-820, Republic of Korea; d Protein Structure Group, Korea Basic Science Institute, Cheongwon-Gu, Cheongju-Si, Chungbuk 363-883, Republic of Korea

## Abstract

This article includes experimental data on the identification of epigallocatechin-3-O-(3-O-methyl)-gallate (EGCG3′′Me) by 2-dimensional (2D) proton (^1^H) NMR analysis and on the information of amino acid and catechin compound profiles by HPLC analysis in leaf extracts of various tea cultivars. These data are related to the research article “*Metabolic phenotyping of various tea (Camellia sinensis L.) cultivars and understanding of their intrinsic metabolism*” (Ji et al., 2017) [1]. The assignment for EGCG3x′′Me by ^1^H NMR analysis was also confirmed with spiking experiment of its pure chemical.

**Specifications Table**

**Table:** 

Subject area	*Chemistry*
More specific subject area	*Food Chemistry*
Type of data	*Table, figure*
How data was acquired	*NMR (700* *MHz NMR for proton frequency, Bruker Biospin), HPLC (Waters HPLC system equipped with a Waters 2996 Photodiode Array Detector)*
Data format	*Raw and Analyzed*
Experimental factors	*Tea leaves were extract in 70% Methanol and in 100% water for NMR and HPLC analysis, respectively.*
Experimental features	*Very brief experimental description*
Data source location	*Division of Food and Nutrition, Chonnam National University, Gwangju 500–575, Republic of Korea*
Data accessibility	*Data are presented with this article*

**Value of the data**

^1^H NMR data provide identifies, structural elucidation and relative quantification of diverse metabolites in leaves of various tea cultivars.

• Epigallocatechin-3-*O*-(3-*O*-methyl)-gallate (EGCG3′′Me) in tea leaves was clearly identified by 2D NMR experiment and was quantified in 1D ^1^H NMR spectrum.
• 2D NMR experiments provide clear structural elucidation of epigallocatechin-3-*O*-(3-*O*-methyl)-gallate (EGCG3′′Me) in tea leaves and thus EGCG3′′Me was quantified by 1D ^1^H NMR analysis.
• HPLC data give information on the catechin-related compounds and amino acids of various tea cultivars and comparable results with ^1^H NMR data.

## 1 Data

**Fig. 1:**
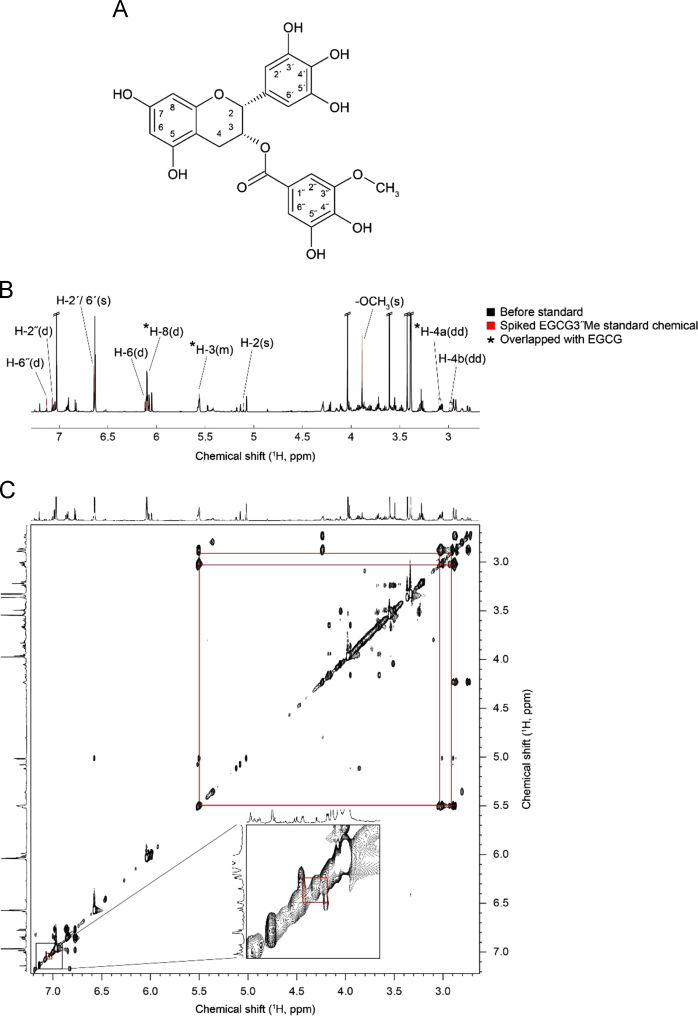
The structural elucidation of epigallocatechin-3-*O*-(3-*O*-methyl)-gallate (EGCG3′′Me) (A) through spiking experiments with the pure chemical (B) and 2D TOCSY NMR analysis of EGCG3′′Me compound in the extracts of EGCG3′′Me-rich tea cultivar (C).

The data include the structural elucidation of epigallocatechin-3-*O*-(3-*O*-methyl)-gallate (EGCG3′′Me) in EGCG3′′Me-rich tea cultivar by two-dimensional (2D) total correlation spectroscopy (TOCSY) NMR experiment and by the spiking experiment of its pure chemical in ^1^H NMR spectrum (Fig. 1) and the profiles of amino acid and catechin-related compound in various tea cultivars by HPLC analysis (Table 1, Table 2).

**Table 1:** The concentrations of catechin compounds and caffeine in the leaves of various tea cultivars used for the present study and harvested in 2015.

Catechin compounds (mg/g dry weight)	Tea cultivars											
	EGCG-rich (Jangwon No.1)			Rich-taste (Jangwon No.2)			EGCG3˝Me-rich (Jangwon No.3)			Wild type		
	1	2	Mean⁎	1	2	Mean	1	2	Mean	1	2	Mean
Gallocatechin	3.55	3.63	**3.59**	1.75	1.81	**1.78**	1.95	2.02	**1.99**	1.76	1.55	**1.66**
Epigallocatechin	57.29	57.90	**57.59**	33.38	33.73	**33.56**	50.87	50.88	**50.88**	51.41	50.81	**51.11**
Catechin	1.17	1.28	**1.22**	1.77	1.72	**1.75**	1.84	1.85	**1.84**	2.04	2.14	**2.09**
Epicatechin	6.75	6.96	**6.86**	7.73	7.77	**7.75**	12.25	12.19	**12.22**	8.77	8.93	**8.85**
Epigallocatechin gallate	55.43	56.46	**55.94**	48.74	48.07	**48.41**	75.30	74.76	**75.03**	50.99	51.70	**51.35**
Gallocatechin gallate	0.55	0.58	**0.57**	0.62	0.61	**0.61**	0.81	0.82	**0.81**	0.62	0.62	**0.62**
Epicatechin gallate	7.75	7.95	**7.85**	11.95	11.72	**11.83**	21.11	20.94	**21.03**	8.44	8.57	**8.51**
Catechin gallate	N.D.	N.D.	N.D.	N.D.	N.D.	N.D.	N.D.	N.D.	N.D.	N.D.	N.D.	N.D.
EGCG3˝Me⁎⁎	N.D.	N.D.	N.D.	N.D.	N.D.	N.D.	7.76	7.68	**7.72**	0.88	0.88	**0.88**
**Total catechins**	**132.49**	**134.76**	**133.62**	**105.95**	**105.43**	**105.69**	**164.13**	**163.46**	**171.39**	**124.04**	**124.32**	**124.98**
Caffeine	15.48	15.56	15.52	18.90	18.62	18.76	26.46	26.20	26.33	20.15	20.13	20.14

N.D. indicates 'no detection'

⁎ Mean values from duplicates of tea leaves mixed from 10 different locations in the tea garden, determined by HPLC analysis.

⁎⁎ The concentrations of EGCG3˝Me in EGCG3˝Me-rich tea cultivar (Jangwon No.3) harvested in 2016 were measured to 10.0 mg/dry weight.

**Table 2:** The concentrations of amino acids in the leaves of various tea cultivars used for the present study and harvested in 2015.

Amino acids (μg/g dry weight)⁎	Tea cultivars			
	EGCG-rich (Jangwon No.1)	Rich-Taste (Jangwon No.2)	EGCG3˝Me-rich (Jangwon No.3)	Wild type
Histidine	6.5968	40.3152	24.44	17.596
Asparagine	12.2512	78.8092	51.4052	70.3836
Serine	359.5288	620	502.4848	475.0896
Glutamine	1.5708	1991.5588	45.7028	114.1688
Arginine	150.314	1378.3888	1508.8392	2854.8336
Glycine	35.026	63.5028	63.4624	34.578
Aspartic acid	338.1676	837.128	824.3584	1052.4808
Glutamic acid	778.534	1740.9808	1813.0348	1674.7828
Threonine	71.3612	157.5832	187.4948	162.762
Alanine	130.89	282.8372	205.662	287.468
γ-Aminobutyric acid	210.9424	214.1856	162.6476	327.1612
Theanine	2115.864	19,678.599	9459.1852	11,744.2456
Proline	28.6912	78.494	37.3932	45.6264
Cystine	18.22	21.6112	63.218	28.8492
Lysine	N.D.	18.494	N.D.	N.D.
Tyrosine	209.7384	213.45	112.5052	166.6496
Methionine	N.D.	N.D.	N.D.	N.D.
Valine	12.8272	40.2452	N.D.	49.4116
Leucine	0.89	11.734	3.9104	11.2532
Isoleucine	19.11	36.182	88.554	21.5856
Phenylalanine	37.1204	75.0264	39.8372	64.348
Tryptophan	99.2148	66.5848	92.0572	157.4424
Total amino acid contents	4636.8588	27,645.71	15,286.192	19,360.716

N.D. indicates 'no detection'.

⁎ Mean values from duplicates of tea leaves mixed from 10 different locations in the tea garden, determined by HPLC analysis.

## 2 Experimental design, materials and methods

### 2.1 NMR spectroscopy analysis of tea leaves

The detailed descriptions of extraction procedure and NMR spectroscopic analysis for proton (^1^H) and carbon (^13^C) are presented in the research article [1].

### 2.2 Liquid chromatography analysis

The derivatization method using AccQ-Tag Derivatization Kit from Waters (Mildford, MA, USA) was applied to the analysis of amino acids [2]. For each cultivar, 1 g of tea leaf powder for each cultivars was extracted with 100 mL of distilled water in a 100 mL-flask by incubating in a water bath at 75 °C for 30 min, and then cooling to room temperature. The filtered extract was mixed with Acc-Tag buffer (140 µL, Waters) and AccQ-Tag derivatization reagent (20 µL, Waters), and left to react at 55 °C for 10 min. After cooling to room temperature, 1 µL of the mixture was injected and separated through the AccQ-Tag ultra column (1.7 µm, 2.1×100 mm, Waters) coupled with PDA detector (UV 260 nm). The separation was performed at 60 °C and for 12 min with the gradient elution, and the flow rate was 0.7 mL/min. The gradient elution (AccQ-Tag ultra eluent A concentrate, solvent A; AccQ-Tag ultra eluent B, solvent B) used was conducted with the filtering and diluting procedure. The gradient conditions were as follows: 0–0.54 min, 99.9% A-0.1% B; 4.75 min, 93.5% A-6.5% B; 7.74–8.5 min,82.5% A-17.5%; 8.7 min, 40.4% A-59.6% B; 8.9–10 min, 99.9% A-0.1% B. With the tea leaf extract, chromatographic analysis for standard chemical such as catechins and amino acids was carried out. The concentration of the chemical in the tea leaf extract was calculated from the calibration curve of the standard chemical integral area.
